# The Ethics Ecosystem: Personal Ethics, Network Governance and Regulating Actors Governing the Use of Social Media Research Data

**DOI:** 10.1007/s11024-019-09368-3

**Published:** 2019-02-07

**Authors:** Gabrielle Samuel, Gemma E. Derrick, Thed van Leeuwen

**Affiliations:** 1 Educational Research, Centre for Higher Education Research and Evaluation (HERE), Lancaster University, Lancaster, LA1 4YD UK; 2 Department of Global Health and Social Medicine, Strand Campus, King’s College London, Strand, London, WC2R 2LS UK; 3 Centre for Science and Technology Studies, Leiden University, 2300 Leiden, The Netherlands

**Keywords:** Evaluation, Governance, Research ethics, Social media, Ethics, Internet research

## Abstract

This paper examines the consequences of a culture of “personal ethics” when using new methodologies, such as the use of social media (SM) sites as a source of data for research. Using SM research as an example, this paper explores the practices of a number of actors and researchers within the “Ethics Ecosystem” which as a network governs ethically responsible research behaviour. In the case of SM research, the ethical use of this data is currently in dispute, as even though it is seemingly publically available, concerns relating to privacy, vulnerability, potential harm and consent blur the lines of responsible ethical research behaviour. The findings point to the dominance of a personal, bottom-up, researcher-led, ‘ethical barometer’ for making decisions regarding the permissibility of using SM data. We show that the use of different barometers by different researchers can lead to wide disparities in ethical practice - disparities which are compounded by the lack of firm guidelines for responsible practice of SM research. This has widespread consequences on the development of shared norms and understandings at all levels, and by all actors within the Ethics Ecosystem, and risks inconsistencies in their approaches to ethical decision-making. This paper argues that this governance of ethical behaviour by individual researchers perpetuates a negative cycle of academic practice that is dependent on subjective judgements by researchers themselves, rather than governed by more formalised academic institutions such as the research ethics committee and funding council guidelines.

## Introduction

Evaluation occurs on a number of levels as a governance mechanism to promote desirable, and regulate undesirable research behaviours. These evaluation-governance gateways ensure that research is conducted responsibly, to a high standard and is aligned to certain standards of behaviour. In terms of governance of human participant research, behaving ethically in research is reinforced through an evaluation network of interconnected actors existing at a number of levels within the academic system and at different points of the research production process – what we call the ‘Ethics Ecosystem’. As an informal, participant-governed network (Provan and Kenis 2007), the Ethics Ecosystem comprises individuals (researchers), organisations (research institutions and the various committees within) and external bodies (publishing houses, funding bodies, professional associations and the governance policies they produce) who participate equally in the promotion, evaluation and enforcement of a shared understanding of ethically responsible research behaviour. This ensures that research is conducted responsibly in a way that is valued by the academy, and minimises risk to participants.

Whilst this informal network ordinarily remains in equilibrium with shared understandings of how to conduct research ethically, there is a risk that the system can become imbalanced when this shared understanding breaks down, for example, when a new research tool is introduced into the Ethics Ecosystem. This paper describes the informal network, and provides some preliminary evidence from the UK on the difficulties the collective, collaborative arrangement is facing in the case of the new research tool, health-related social media (SM) research. In particular, it provides evidence that this new research tool has led to imbalances in the ecosystem. Our work stems from a pilot project funded by the UK Wellcome Trust, which was designed to explore ethical decision-making in the field of SM health-related research. Health-related SM research is likely to be particularly ethically sensitive due to the personal nature of the information shared, and the more obvious risks to personal identities and profiles, and so offers a good case study for analysis.

In particular, this UK pilot exploration of health-related SM research at a meta-level has identified some evidence of the application of a form of “personal ethics”. When applied by researchers, this personal ethics approach may have the potential to decrease the effect of governance of other actors in the UK Ethics Ecosystem and we discuss the implications of this.

### Social Media Research

For the purposes of this paper, SM research is defined as research which uses data sourced from any social networking site including, but not limited to: Twitter, Facebook, YouTube, blogs, and password-protected and non-password-protected chatrooms and forums. In terms of research, this can include large quantitative data mining/modelling methods through to more qualitative in-depth analyses. SM research provides a seductive new methodological tool that has the potential to reveal new insights into information sharing (Williams et al. 2015), policy discussions (Campbell 2009), and online behaviour in general (Panzarasa et al. 2009). In the instance of health research, SM sites such as Facebook, Twitter and online forums are seen as particularly rich sources of data (Vayena et al. 2012), as an effective way to recruit a large number of participants (Chu and Snider 2013), as intervention platforms for specific health conditions (Renton et al. 2014; Rice et al. 2014), and as a general source of seemingly publicly available data (Gabaron et al. 2014). Examples of SM research applications include Twitter data being used to track epidemics, and blogs and online platforms being used to explore health behaviour and experiences of people living with health conditions (Aramaki et al. 2011; Wilson et al. 2015).

There are a range of ethically contentious dimensions related to SM research, and the ongoing and complex nature of this research has been suggested to be potentially challenging for researchers and ethics committees (Zimmer 2010; Henderson et al. 2013). Conceptually, much headway has been made towards defining the range of issues associated with SM research. Given the diversity of the field, this work has arisen from a variety of disciplines including, for example, sociology, computer science, media/communications studies, health research and allied fields, anthropology and bioethics. Two primary concerns have emerged: whether to classify SM research as human subjects’ research or text-based analysis (Solberg 2010; Herron et al. 2011; Markham and Buchanan 2012; Solberg 2012; Convery and Cox 2012; Lomborg 2013; Hudson and Bruckman 2004; Bassett and O’Riordan 2002; Grinyer 2007); and the issue of what constitutes public and private spaces (Abril and Cava 2008; Herron et al. 2011; Vayena et al. 2012; Convery and Cox 2012; Markham and Buchanan 2012; Snee 2013; Chiasson et al. 2006; McNeilly et al. 2013). Indeed, it has been claimed that ‘*people in public, online environments often act as if these environments were private*’ which can result in participants feeling violated if studied without their awareness. Such concerns raise questions about whether consent should be received for SM research to proceed, and under what conditions (Hudson and Bruckman 2005: 298). This is thought likely to depend on factors such as the particular group of participants being studied (Eysenbach and Till 2001) and/or the sensitivity of the topic under discussion (McKee and Porter 2008, 2009), with no present consensus. This ambiguity means that as SM researchers strive to act ethically responsibly, an ethically grey area for promoting ethically responsible behaviour and dissuading unethical research behaviour remains. This risks differing interpretations for ethical behaviour being practiced at each Ethics Ecosystem governance level - by different researchers, research groups, research institutions, funding councils, publishing bodies, and nations.

**Fig. 1:**
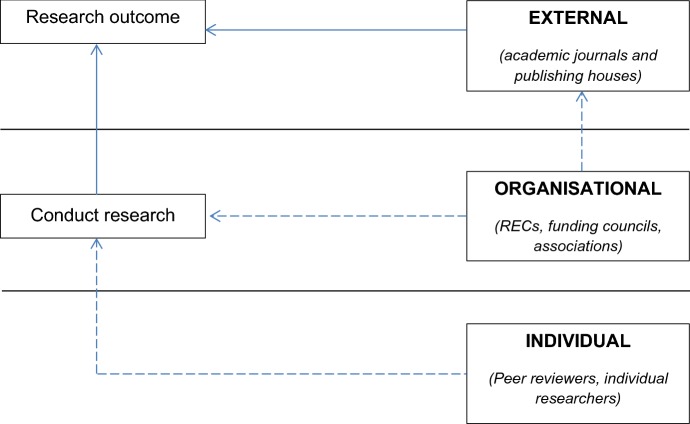
The ethics ecosystem: levels and interactions of research governance

By using UK health-related SM research as a case study, this paper explores what may happen to the Ethics Ecosystem when there are differing interpretations of ethical behaviour within a specific field of analysis. It preliminary analyses the disparities and conflicting norms of best practice that have emerged between the different actors within the UK SM research governance Ethics Ecosystem. To do this it uses a multi-modal research design which explores ethical approaches to SM data use at different stages of the research process from inception to publication, including at the level of researchers, funding bodies, research institutions, and publishing houses. A full schema of the main UK ecosystem governance actors integral to this study is offered in Fig. 1. Our findings provide some evidence that there may be a lack of community consistency, fostering a culture in which decisions about the ethical use of SM data is primarily made by a reliance on individual researchers implementing a form of “personal ethics”, rather than by a shared norm around the use of SM data by actors within an overarching UK Ethics Ecosystem. By examining the promotion of ethical research behaviour on the network-level rather than individual actor-level, this paper provides preliminary insights into how, if the ‘personal ethics’ approach is indeed widespread, this informal system can work more efficiently and collectively in the UK as more, new and ethically complex research tools emerge.

### Evaluation as a Network Governance Mechanism

Although “governance” is a contested and highly ambiguous term (Jordan 2008), it is used here as a notion of how society or an organisation is ruled beyond formal institutions and processes (Molas-Gallart 2012). The consideration of how actors work collectively, rather than individually to govern and regulate behaviour within a network using stages of evaluation is relatively under-developed (Provan and Kenis 2007). Further, the consideration of ensuring that research is conducted responsibly and ethically as a factor of a larger, informal, participant-led network is particularly innovative. In this consideration, the regulation of ethical behaviour is not seen as the result of a hierarchical system, but instead as one that is pluricentric. Whilst this network is not formally ‘governed’, the practice of evaluation performed by each actor acts as a gateway to filter out behaviour that is considered as external to the shared norms and trust of the network. As an example, researchers will follow practices they see in publication and reviewers will demand what other reviewers demand of them, thereby saliently and self-reinforcing behaviour that is considered by all actors as within the norms of acceptable research behaviour.

This, we argue in this paper, is the case of the Ethics Ecosystem (Fig. 1) that, as a network and a series of evaluation gateways, acts to promote self-constituted norms relating to ethical research behaviour. This system of governing ethical research behaviour has been expanding over the past half a century, originated out of a series of atrocities relating to a misuse of human participants (Truman 2003; Boden et al. 2009; Stark 2011), and relates to a desire to normalise personal ethical barometers regarding how to conduct human participant research, specifically in relation to respect for persons, justice and beneficence.

However, problems occur when the network encounters a new, ethically ambiguous tool such as with the rising prominence of research using SM data. Since the network acts pluricentrically and informally, it is difficult for individual actors to approach the ethical considerations of this tool equally. Therefore, there is a risk that each actor applies their governing evaluation differently, resulting in a decrease in the efficiency of the network and the disintegration of the previously cooperatively applied norms regarding what is collectively agreed to be ethical behaviour. In addition, by reducing the efficiency of the system as a whole, there is a risk of researchers developing and applying a degree of ‘personal ethics’, thereby dropping out of the network’s governing cooperation entirely. This reduces the ability of the network to govern and promote ethical behaviour relating to SM research data use, as well as to develop new shared norms to apply in future situations for SM research data use.

### The Ethical Ecosystem: Individual, Organisational and External Governance

Some authors have noted that research ethics committees (RECs; Institutional Review Boards (IRBs) in the US) hold most of the power in determining ethical norms of practice within the academy (McAreavey 2013). Others disagree, arguing that ethical behaviour is reinforced by the interaction of a number of actors within the academy, and a range of positions and voices influence conceptualisations of ethical practices (Cannella and Lincoln 2007). In line with this, this paper argues that all these actors are together in an informally governed, participant-led network we term the ‘Ethics Ecosystem’. This Ecosystem is large and complex, working at the individual, organisational and external level of the research process to promote and enforce a community-wide understanding of ethically responsible research behaviour. And whilst some actors within this ecosystem may be perceived to have more power than others in regulating ethical norms (e.g. the RECs), each actor has a role to play: within this ecosystem and through a series of interconnections, actors all rely on each other to govern and encourage ethical research behaviour, as well as dissuade unethical behaviour through a variety of evaluation-governed gateways. This network works if, as is in the majority of cases of participant-led networks (Provan and Kenis 2007), the norms and definitions of ethical behaviour are shared by each actor. In other networks described by Provan and Kenis (2007), for example, those of many health and human services as well as in movie production and co-operative buyer-supplier manufacturing models, behaviour is enforced through a formal regulator mechanism. However, for participant-led networks, a shared commitment to the goals of the network as well as compliance that is ensured through trust and obligation, results in the informal application of rules and norms governing behaviour that become self-constituted and accepted over time. Informal regulatory mechanisms for research ethics are applied by different actors within the network as a range of disciplinary codes of conduct.^1^ The principles of these informal mechanisms are familiar to researchers, RECs and publication outlets alike as a collectively shared network-norm. The norms contained within these codes, if understood similarly, become standard practice that is re-enforced through evaluation-governed gateways, thereby informally regulating research behaviour. Below we discuss each level of the Ethics Ecosystem, highlighting how each group of actors have the power to shape ethical norms and influence ethical behaviour.

At the first level of this system, individual researchers are responsible for developing research plans and deciding (when organisational guidance is ambiguous or not mandatory) what areas of this plan require ethical clearance before the research has begun. At this level there is an underlying assumption that researchers act with both intellectual and ethical integrity that is separate from the ecosystem of complex governance – for example, in their relationships with research stakeholders, including participants, to personally ensure that no ethical boundary is overstepped during the course of the research. Some feel that governing behaviour at this level is the sole-responsibility of researchers in line with their own understanding of ethics (personal ethics) that is in line with the definition of professional practice. McAreavey (2013) even goes so far as to suggest that researchers should ‘reclaim’ research ethics as an inherent component of the professional practice of research, and redefine their behaviour due to their own definition of ethical behaviour (McAreavey 2013). This idea is shared by Boden et al. (2009) who warn that over-bureaucratic regulation and checking of ethical behaviour by other actors within the Ethics Ecosystem, as well as by a network governing ethical behaviour as separate from professional research behaviour, risks the application of an unacceptable level of power to halt scientific progress based on the mask of ethics regulation (Boden et al. 2009).

Funding bodies and RECs constitute the next, organisational level of the Ethics Ecosystem. In the case of the former, their ‘ethical’ power lies in their choice to fund, thereby indirectly endorsing certain ethical behaviour within applications, or not if the intended research behaviour is not perceived to be suitably in line with commonly shared norms regarding ethical behaviour. In terms of the latter, RECs play an important gatekeeping role with the goal of controlling research behaviour (Molas-Gallart 2012) by expecting researchers to submit research plans for peer-based review to enforce community norms regarding ethical behaviour. Here, ethically responsible behaviour is defined in terms of the perception of ‘risk’ to both research participants, as well as to the organisation. This sometimes leads to a clash in priorities, and because of this, scholars have argued that this level of ethical scrutiny is ‘overly bureaucratic’ (McAreavey 2013) and ‘hijack[s] research ethics’ through a system of research management. Moreover, they claim that researchers who act as peers within RECs operate under a ‘*pre-determined framework…set by central university management*’ (McAreavey 2013; Tierney and Corwin 2007) and do not take the subtleties of group evaluation process into account.

On the final, external level of the ecosystem are the academic journals and publishing houses, which can insist authors to declare prior ethical approval and/or ethical considerations during the peer review process, without which the article will not be published. In this way, they have the power to ensure that community-wide norms of ethical behaviour have been adhered to and are therefore gatekeepers in more than one sense (of research and ethical standards). With the future careers of individuals based heavily on their ability to produce research outcomes, the cost of non-compliance is potentially very high. In addition to their own use of evaluation, these actors also rely heavily on the trust that ethical behaviour has been adhered to, and formally endorsed at both the individual and organisational levels.

At the different levels within this ecosystem, trust in each actor’s ability to check and enforce commonly held norms governing ethical behaviour by all actors is central (Hedgecoe 2012). Tensions, however, occur when there is a conflict between the personal (‘individual’) interpretation of ethical behaviour around one form of data, with more formalised forms of ethical scrutiny (‘organisational’) and research management. Within a system where actors hold conflicting norms, or are unsynchronised with their experience of a specific research tool to the extent where norms of behaviour have not yet formed, there is a real risk that the lack of consensus leads to the formation of ‘individual’ resentment, towards ‘organisational’ level governance, or worse, norms that inadvertently encourage unethical behaviour, and lead to greater risk to research participants. McAreavey and Muir (2011) highlighted the increasing alienation of social science researchers from the restrictive, unmovable REC and argued that this alienation led to serious consequences for the ethical standards of social science research, thereby promoting the use of personal ethics, where researchers self-assessed and governed their own ethical research behaviour (McAreavey and Muir 2011). Differing degrees of opposition to formal organisational level governance over ethical behaviour has also been reported in similar studies and works (Hammersley 2009; Cannella and Lincoln 2007; Van den Hoonaard 2016). It is therefore important that organisational ethics actors ensure that their governance is in line with the norms exercised by other actors within the ex-ante evaluation system, as well as up to date with the research, and methodological tools in use within the research system.

Such is the case with SM research. When using SM data for research, ordinarily shared norms of ethical behaviour are obscured because such research blurs the ‘fundamental rights of human dignity, autonomy, protection, safety, maximisation of benefits and minimisation of harms’ (Markham and Buchanan 2012: 4). This leads to different interpretations by different governance actors (individual, organisational, external) about common ethical principles such as risk to participants, whether there is a need for consent, and whether to consider the data as publicly available (Hibbin et al. 2018).

Several initiatives have also been developed to aid SM researchers with their ethical decision-making. To name just a few, in the US, Larsen and colleagues have created a tool for SM researchers and research ethics committee members which draws on participatory approaches and shared experiences to grapple with SM ethical issues (Torous and Nebeker 2017); internationally, scholars have established a database of case studies at Sage Publications designed to help more clearly understand abstract methodological concepts in practice (SAGE); a range of UK workshops have been convened on the topic and special issues published in the literature (Sormanen and Lauk 2016); and finally, New Social Media, New Social Science (NSMNSS) - a collaborative UK network of researchers and stakeholders in the field - has been established to facilitate and engage discussion in this area.

A number of ethical guidelines have also been published for SM research. The most prominent of these - those proposed by the Association of Internet Researchers - suggest a series of comprehensive questions for scholars to ask themselves prior to embarking on research (Markham and Buchanan 2012). This non-prescriptive strategy has been replicated by the guidelines of various discipline-specific professional bodies, research groups and institutions - both for academic research, and outside the field (Townsend and Wallace; ESOMAR 2011; Jones 2011; van Wynsberghe et al. 2013; Social Media Research Group 2016; British Sociological Association 2017). Guidelines with a specific health emphasis include those from the British Psychological Society (British Psychological Society 2017), and a more recent sub-section of the CIOMS guidelines dedicated to online research (Council for International Organizations of Medical Sciences (CIOMS) 2016). Some of these guidelines are relatively comprehensive, stressing the importance that researchers consider the validity of their work (do the methods answer a useful question?); the legal implications; the implications to social media user privacy, including in terms of data storage; and issues concerning the traceability of using de-identified quotes. Some even adopt a detailed and useful case study approach to illustrate ways to proceed ethically when faced with different topics of social media study, data collection methods and types of analysis (Townsend and Wallace). Even so, the guidelines still remain thoughtful considerations rather than ethical obligations to be implemented. This, say some scholars, is vital, because whilst some empirical work has explored SM user perceptions about such SM research practices (Hudson and Bruckman 2004; Taylor et al. 2014; Vayena et al. 2012; O’Connor 2013; Mikal et al. 2016; Beninger et al. 2014; Evans et al. 2015; Williams et al. 2017), this research is still too limited to fully acknowledge the nature of potential harms which may or may not exist for users (Weller 2015). Whilst this may be true, at the moment, there is some suggestion that such non-prescriptive guidelines are less than adequate (Woodfield et al. 2013).

Using a multi-methods research design, this article explores the UK health-related SM research Ethics Ecosystem and, in particular, how this governance system plays out in practice in the field of SM research where so many differing interpretations and thresholds for ethical behaviour prevail, and most guidelines and initiatives are non-prescriptive in approach and therefore do not tell researchers how to act. Further research will explore SM research governance in other non-UK countries. The study is a pilot project and does not aim to be representative or make broad generalisations. Rather, it aims to generate hypotheses which can be tested in further research. Having said this, we do use our findings to make recommendations about SM research ethical governance, which we feel may improve the current lack of shared understanding evident not only in our findings, as we shall show, but also in much of the literature, as discussed above.

## Methods

A mixed methods analysis combining guideline analysis, bibliometrics and qualitative (interview) techniques was employed in this study. This multi-modal perspective enabled the study to gain a multi-stakeholder understanding of the various levels of research governance and their approach to the ethical use of SM data. To this end, perspectives from published SM researchers, university REC members, publishing houses, peer-reviewed journal editors and the UK funding councils were gathered using relevant quantitative and qualitative approaches. Professional Associations were not included in this analysis. A summary of the data types, its nature and its source are included in Table 1.

**Table 1:** Sources and nature of data used in this study

Ethics ecosystem actor	Nature of methodological inquiry	Source
Research ethics committee	Interviews (qualitative)	REC members
Universities	Website analysis and clarifying survey	University ethics managers
Researchers	Interviews (qualitative)	Bibliometric search of authors of SM articles
Publishing houses	Document analysis and clarifying survey	Selective sample
Journals	Document analysis and clarifying survey	Bibliometric search for journals publishing SM articles
UK funding councils	Document and website analysis	All RCUK actors

### Research Ethics Committee Members (Interviews)

Chairs and/or members of university-level and departmental/faculty-level RECs of 20 research-intensive universities in the UK were invited to participate in an interview. In total, 63 individuals or generic ethics committee email addresses were contacted to request participation in the project, and 19 were interviewed (19/63; 30.1% response rate). Participants represented 13 UK institutions across 18 different university-level or faculty/department-level RECs (science, information science, social science, humanities, medical or health science and psychology). They included nine REC Chairs, one deputy Chair and nine REC members. Ten participants sat on a university-level REC (which covers all research faculties), and 13 participants sat on a departmental/faculty-level REC (four participants sat on both).

Interviews were conducted by GS either face-to-face, over the telephone or via Skype lasting between 40-60 minutes, and were digitally recorded. The interview schedule was broad, exploring interviewee’s views about the ethical issues surrounding the use of SM data for research; their views about whether such research should require ethical approval, and knowledge about the policies at their own institution in relation to this; their experiences of reviewing such research in a REC capacity and their decision-making in relation to this; any guidelines, training or literature they had used in order to aid their decision-making in this area; and, for those with no experience in this area of ethical review, how interviewees thought they would make decisions about this research in their capacity as a REC Chair/member.

### Researchers (Interviews)

An in-depth bibliometric search for publications using SM research data originating from the UK (described below) was conducted in order to identify UK researchers using SM data (n=147). This data was manually cleaned to select those publications specifically reporting the use of SM health data (n=27). SM researchers were invited for interview. A total of 14 researchers participated in the interviews. Scholars were from the fields of psychology, computer/data science, informational systems, STS, anthropology, linguistics and public health; and were experienced with using a broad range of different qualitative, quantitative and data modelling methods. Data saturation was reached for the themes described within this paper.

Interviews were conducted by GS over the telephone or via Skype and were digitally recorded. The interview schedule was broad, exploring interviewee’s views and experiences about the ethical issues surrounding the use of SM data for research and their decision-making in relation to this; their views about whether such research should require ethical approval, whether they choose to have their research reviewed, and knowledge about the policies at their own institution in relation to this; and any guidelines they referred to in order to aid their decision-making in this area.

### Analysis of Interviews (Researchers and REC Members)

We understand our interviewees are self-selected and this may mean there are biases to our research data. However, the aim of this analysis is to generate hypothesis rather than create a representative sample from which we can generalise.

Analysis of interview data was approached using inductive reasoning, employing the inductive approach of grounded theory (Glaser and Strauss 1967). The analysis (or coding) of data was based on two inter-linked rounds: overview analysis and detailed analysis (Glaser and Strauss 1967). Overview analysis consisted of memo-making and broad coding. Extensive memo-making was employed by the interviewer directly after each interview. Broad coding proceeded by scanning the interview transcripts for relevant ideas and themes before each of these themes were explored in a detailed analysis.

### Universities

We employed two modes of data collection. First, university intuitional webpages were searched for information relating to ethical practices about the use of SM data for research. Second, to confirm our online search of university webpages, institutions were contacted via email to request whether (a) their institution had published any ethical guidelines for researchers using SM data, and (b) such research at their institution required ethical approval. Follow up telephone calls were conducted for non-responding institutions.

### Publishing Houses, Journals and UK Funding Councils

Information about the development of ethical guidelines, processes and practices relating to using SM data for research was sampled from UK universities, UK funding bodies, publishing houses and journals. The sample included the top 20 UK research-intensive universities, as defined by the UKs national assessment exercise, the Research Excellence Framework; the main UK bodies who fund research, including Research Council UK and its umbrella organisations, the Wellcome Trust and the National Institute of Health Research; the top 5 most prolific publishing houses, which account for more than 50% of all papers published,^2^ including Reed-Elsevier, Springer, Wiley-Blackwell, Taylor and Francis, and Sage; and the top 10 most common journals plus the top 10 most common health-related journals which publish SM research (n=16, excluding overlaps). A bibliometric search was performed (described below) to identify publications originating in the UK that had utilised SM data, and the journals that had published these articles (n=2639).

Similar to the method employed for searching university ethics guidelines, we employed two modes of data collection. First, we navigated webpages of the funding bodies, publishing houses and journals searching for any ethical guidelines related to the use of SM data in research. Second, emails were sent to each of the relevant institutions requesting information about this. Emails to non-responding institutions were followed-up with a telephone call for funding bodies and publishing houses, but not for the journals (responding journals n=11/16 including 6/10 most common journals and 7/10 most common health-related).

### Bibliometric Search and Identification of SM Research

CWTS maintains an in-house version of Web of Science (WoS) that includes publications from 1981 to the present. Within this database system, three complementary datasets were used to identify SM relevant articles, journals and authors;

1. *Dataset of terms (and combinations of terms) used in titles and abstracts of publications indexed by WoS.*

Here, meaningful information is extracted from the title and abstracts using a linguistic parser. This parser creates relevant noun phrase groups, which include words observed in close proximity in a title or abstract (van Eck et al. 2010).

2) *Dataset of institutional addresses.*

This dataset contains addresses from knowledge producing institutions. These addresses have been cleaned to eliminate small errors in name giving and to unify and standardize the variations under one main umbrella (Waltman et al. 2012).

3) *Dataset of author names.*

This database consists of a number of sets through which author disambiguation is organized. It links publications to one single person, by using both bibliographic as well as bibliometric information (Caron and van Eck 2014).

Using the first dataset, we delineated social media through noun phrase groups in both titles and abstracts. This included two sets of words; one that contains clearly social media related notions (e.g. blog, Facebook, Instagram, internet research, Pinterest, social media, Twitter, web forum, and YouTube); and a second that noun phrase groups that relate in some sense to online research or facilities (e.g. online chat, online comment, online comments, online communities, online community, online discussion, online discussion forum, online discussion group, online discussions, online forum, online group chat, online group discussion, online research, online space, online support, online world). No distinction was made between general terms, and terms that relate to products or platforms in this first list.

Initially, occurrences were found for both sets by using a fuzzy search method, which would then also include wild card searching, which means truncating words, thereby allowing some wider variety of occurrence of soft terms in the final search results. This led to a very high recall, and a high level of noise within the data. To rectify this shortcoming, searches for the exact occurrence of the term were performed. This would result in terms used in the second set that occur in both single and plural variations (e.g. combinations of online with comment/comments). As such it was important to establish this relationship between the term and the publication where the term was included, in order to collect a more relevant list of SM publications.

After the first datasets based upon these two sets of noun phrases were constructed, terms to the second dataset described above were linked to contain an address in the United Kingdom only. From this recall, author information was linked to contact information in order to isolate those SM topic publications with a UK address (n=2639).

## Results

### Individual Level Governance: Researcher Views

Researchers’ experiences made them acutely aware of the various ethical issues associated with using SM approaches in their research. They were also familiar with various SM ethical guidelines published to help them negotiate these ethical issues, most prominently those proposed by the Association of Internet Researchers and the British Psychological Society, though in line with previous reports these were generally viewed as not overly helpful (Woodfield et al. 2013). This was because the adoption of a researcher-led, case-by-case approach meant that these guidelines often acknowledged the messiness and complexity of SM research, described the ethical issues, and suggested ethical questions to ask one self, but did little to prescribe how to act. Researchers described them as ‘*deliberately grey’* (researcher 1), feeling that they were left with little instruction on how to approach their research ethically: *‘those ones (guidelines) and the BSA, I think those are the only two. They weren’t very…they weren’t kind of guidelines are they, they’re more like make your own decision’* (researcher 6). The speed with which SM research was progressing also meant that guidelines were being updated regularly - and that some practices deemed ethical in one set of guidelines could be considered ethically unreasonable in further revisions of the same guidelines. As interviewee 12 noted in relation to some research they had conducted, it was ‘*actually in line with those [discipline and university associations research policies for dealing with online data] but only because [they] were quite underdeveloped at the time. But I think since then those policies have been revised’* (researcher 12).

This lack of any prescriptive standard, alongside the need to adopt a researcher-led approach, gave interviewees permission to approach ethical decision-making in terms of how they felt ‘personally’ about their ethical choices, and the types of ethical issues which were salient to them within their own work: *‘there’s a series of ethical dimensions and considerations and it is up to each researcher to reflect on that and consider what they mean for their particular project for their particular research question and population and specific methodology’* (researcher 3). Interviewees thus placed emphasis on the subjective, individual nature of ethics when justifying their research practices. We can see below how, drawing on the subjective nature of ethics as key, interviewee 5 followed their own ethical guidelines when thinking about the ethical issues associated with their research:

Interviewer: Are there any guidelines in particular that you follow in your own research?

Researcher 5: It’s my guidelines. Everybody has their own definition of ethics….

And interviewee 10 spoke about how any researcher, with any subjective beliefs about ethics, could justify their research just by ‘working around’ the vague guidelines: *‘they are [guidelines] slightly contradictory in places and you can argue around them…so there is some issues around integrity that don’t necessarily hold true and you can argue either way for some of the issues around that’.* This meant that the ethical weight placed on any particular issue within a particular research project varied amongst interviewees. Ethically salient for the above interviewee 5 was that research should not be exploited, but rather should be performed to provide benefit to society. This meant that this interviewee paid particular care to the choice of their research topic:

I question myself that some of my research could be used in other applications that may not be ethical enough…but that’s part of research, people can mine text and do that for different reasons, and that’s the only question I pose to my work… I think I make strict restrictions to me and my collaborators about being ethical…I value more things that will benefit society….so that’s my main guidelines (researcher 5).

In contrast, for interviewee 4, ethical weight was placed on decisions relating to the protection of SM user privacy. In the following quote, and by comparing the different approaches used in two different papers (one which drew Twitter data from individuals with many followers, and the other which drew Twitter data from individuals with less followers), this researcher draws on their own moral compass to make judgements about when and when not to expose the identities of particular Twitter users:

In some sense it was an ethical issue because I didn’t really want to identify people, because I just didn’t really think it was relevant to the point I was making in the paper. Whereas in the first one…[other paper] I wanted to bring them [Twitter users] out as clear, individual[s] with large numbers of followers. I also kind of feel that when you are presenting someone with a very big following on Twitter, they are unlikely to feel that their privacy is being invaded by being the subject to the research…. I didn’t feel any ethical responsibility to protect their identities because their identities were really what I was interested in (researcher 4).

This comment also raises interesting concerns relating to the need for privacy and de-identification of Twitter data in all instances of research and the different methodological lenses which are used when approaching different research questions. Finally, for interviewee 9’s subjective ethics, the issue of using freely accessible data irrespective of how SM users would feel about it was viewed as ethically reasonable because for this interviewee, what was analogous to that which was deemed reasonable in past situations, was morally acceptable in the present:

I mean Anne Frank’s diary immediately comes to mind….she didn’t intend that to be public…so there are new challenges…but in terms of getting new data that’s publically available and using that for research where the data wasn’t intended for research, I think that’s not fundamentally new…[and] yes, perhaps that’s, yes [reasonable in an ethical sense] (researcher 9).

With such a personal approach to ethics, researchers’ ability to justify their ethical choices to both other researchers, as well as, where necessary, to ethical review boards became a key priority: ‘*I think that it’s more a fence of guidelines and researchers having leeway in the way they put those guidelines into practice and being prepared to justify what they’ve done’* (researcher 2); *‘there is a sense of you got to develop the sense of what’s right here, be the expert in that and then put that across and you know, make your case’* (researcher 7). This seems reasonably justified as an approach to ethical decision-making in a complex field, and indeed, one prescribed by the various guidelines available. However, comments by some interviewees suggested issues in terms of the heavy burden of decision-making placed upon themselves (‘*it does feel like that for everyone…they sort of have to battle with it themselves and be happy with themselves that they’ve got some justification’* (interviewee 10)). In addition, there was some suggestion that placing the burden on the researcher could lead to poor ethical decision-making. For example, interviewee 12 explained how during their observational study of an online mental health support group they had not gained SM user consent but rather, to maintain user privacy, had only observed those SM platforms whose data was unsearchable on Google. This interviewee realised in retrospect that failing to ask SM user permission might not have been ‘a great case study in ethical practice’:

The fact that the people that organised the site had made it so that it didn’t show up in Google search results suggested that they had a high level of concern for privacy…out of the public eye by making them not show up on Google. I should have been conceiving over those forums as kind of highly private and hence places where a higher level of ethical concern to the participants should have been adopted really. So, yeah, I mean it’s probably not a great kind of case study and ethical practice…I’m embarrassed about it…

This situation arose because of the little institutional guidance and policy at the time to guide this interviewee about the best course of ethical research and the fact that their colleague ‘*was kind of happy that as long as it [the research] was in line with the university’s policy then that was alright…*’ i.e., if no policy existed on gaining consent, then there was no need to worry about adopting this approach during the research project. Personal ethics in this instance meant that this colleague could turn a blind eye to key ethical issues on the basis of vague or lacking policy-making.

In summary, even though all interviewees spoke about upholding the highest forms of ethical standards, because of the ethically grey area surrounding SM research, because there are no shared norms of practice, there was a variation in the way in which researchers ‘practiced’ ‘being ethical’, which we refer to as a ‘personal ethics’ approach.

Different Practices in Terms of the Need for Ethical Approval

A ‘personal ethics’ approach may also lead to inconsistencies in terms of whether researchers felt the need to receive ethical approval for their research^3^. For some, all research should be subject to ethical review ‘*in some form’* (researcher 7), since *‘it’s still research, so I would say anything that involves – even if it’s looking at newspaper coverage…should still be subject to ethical approval’* (researcher 2). Other interviewees did not feel the need to gain ethical approval for their research: ‘*for data scraping I think no [I would not get ethical approval]’* (researcher 9).

Differences in practices were not necessarily relevant to how ‘ethically-minded’ interviewees were, nor was having ethics approval viewed as a proxy for good ethical practice, since *‘you can be ethical in how you approach the data, how you talk about the people who join the sites. So there’s ways of being ethical but that’s not actually anything to do with getting through an ethics committee approval’* (researcher 1). Rather, the choice of whether to gain ethical approval for research related more to interviewees’ personal perceptions about the type of research methodology or analysis under question. One criterion related to how aggregated the data was during analysis, since for many (though not all) interviewees’, the aggregation of data meant that ethical approval was unnecessary: *‘it was not the requirement so far because we did not collect user level, let’s say private data but mostly aggregation of tweets, public data*’ (researcher 5). Another criterion reflected interviewees’ perceptions about whether SM data was viewed as text, analogous to any media text, or as human participant data: *‘in my institution we don’t [have to go through approval] at the moment….why I think I can do this without the university approving because that is not actual empirical data…[…]…we aren’t talking about the person, we’re talking about produced text’* (researcher 1).

Our data suggests at least limited evidence that these different personal perceptions towards governance and practice stemmed not only from a variation of ‘personal ethics’, but also from different philosophical, epistemological, and normative frameworks associated with each specific discipline and mode of analysis (Trevisan and Reilly 2013; Ostman and Turtiainen 2016): ‘*this is where to me ethics is entirely bound up with the nature of your research question and the philosophical stand point that the research has’* (researcher 4). For example, through a discourse analyst lens, SM data was viewed as text and interviewees adopting discourse approaches did not perceive a necessity to seek permission to use SM data, nor was ethical approval viewed as pertinent:

I’ve certainly had some conversations where people say it has to be human participants because it’s people generating conversations with each other when they’re living with illness… So I think different people will look at it in different ways….as a methodology, I’m just looking at text. It’s text about the human experience… (researcher 3).

For an anthropologist we spoke to, identity was something very different. Interviewee 6 spoke about the relationship formed between a researcher and SM user when a researcher reads, interprets and analyses SM user experiences. For this interviewee, this relationship amounted to a requirement to receive permission to conduct the research:

The data you use is created by someone else in the interview interaction, you’re both creating it together and you have accountability to the other person and a responsibility to the other person. You’re told about the reciprocal relationship…I saw these [online data] as personal accounts, I didn’t really see it as data or text… that’s probably because of my background….[…]…I went back to my interviewer feeling and thought I wanted to present it as participants…. (researcher 6).

For one computer scientist mining data for aggregation and data modelling, data was generally thought of as usable without the need for any form of permission: ‘*the idea of just grabbing the data, maybe six months, maybe potentially years after it was written, I don’t think that requires ethical approval or necessarily the consent of the people involved’* (researcher 9). Though this was not always the case for computer scientist interviewees, and researcher 13 explained their views on getting ethical approval for research on aggregate data: *‘if in that aggregate data set it begins to emerge that “oh look there is lots of comments about Dr X”…at least if we’ve done it under ethics approval we’ve got to the starting point and framework and we know how we should behave in regards to that data’*.

Overall, this section has shown that our interviewees adopt a ‘personal ethics’ approach to decision-making about SM research. We re-affirm that calling it a ‘personal ethics’ approach carries no moral judgement since all interviewees demonstrated to be incredibly reflective in terms of how to approach ethical decision-making for SM. Rather, because there were no shared norms of practice for SM research, interviewees had to rely on their personal beliefs on ethical practice meaning that a ‘personal ethics’ approach emerged. The issue with adopting this approach relates to its consequences. One consequence highlighted by our findings is the inconsistent viewpoints in terms of whether researchers should or should not subject their research to ethical review. Moreover, below we show how an inconsistent and under-developed institutional governance of SM research at other levels of the Ethics Ecosystem sets up the potential for some of the personal ethics viewpoints to feed into practice, which could be problematic if (in the worst case scenario) a researchers’ personal ethics approach is unethical. This was the case for at least one interviewee.

### Institutional and External Level Governance: UK Funding Bodies and Research Institutions

Document analysis showed that the UK Economic and Social Science Research Council (ESRC) was the only UK funding body with any concrete guidelines for research which uses SM data (unsurprisingly given its social science remit) (Orton-Johnson 2010). These guidelines provided an overview of the ethical issues related to the use of SM data for research and stressed that ‘*all ESRC-funded research must be subject to an appropriate ethics review*’.^4^ All other funding bodies provided little in the way of guidance for research in this area. Rather, they placed the onus on researchers and/or research institutions to adhere to ‘*the highest level of research ethics, in line with requirements set out by national and international regulatory bodies, professional and regulatory research guidance and research ethics frameworks issued in appropriate areas’* (Research Councils UK: 3). These findings have been confirmed elsewhere (Taylor and Pagliari 2017). Regulatory mechanisms at Higher Education institutions were varied, with some universities requiring all research using SM data to undergo an ethical review process (n=7/17) and others requiring approval only for research drawing on SM data outside of the ‘public arena’ (n=4/17). Five universities assessed on a case-by-case basis, and one institution placed responsibility upon the researcher themselves to more generally determine whether their research required ethics approval following the completion of a checklist.

The variance in institutional policies points to an inconsistency of ethical decision-making between UK institutions in terms of which SM research projects require ethical approval. This inconsistency alone is enough to suggest that over-arching policies at present are inadequate – and we later argue in our discussion for a more uniform approach to this. Though the main point we wish to note here is that when institutions do not demand ethical approval, or when researchers opt to by-pass the process as shown in the above section, researchers’ ‘personal ethics’ approach to their research is not subject to ethical review until the point of publication (we come back to the point of publication later on). For researchers who do choose/are required to subject their research to REC scrutiny, REC members’ limited experience with reviewing proposals which draw on SM methodologies meant that they often turned towards researchers’ ‘personal ethics’ to aid with decision-making.

### Institutional Level Governance: Research Ethics Committee Members

A detailed analysis of our REC interviews has been discussed elsewhere (Hibbin et al. 2018; Swift et al. 2018). In short, our REC member interviewees had different views about the nature of SM data, the risk SM research presents to SM users, and whether researchers need to receive consent from SM users before research can proceed (Hibbin et al. 2018). Moreover, whilst REC members were increasingly aware of the types of ethical issues associated with the use of SM data in research, much inexperience remained with relation to reviewing research proposals during the ethical approval process (Swift et al. 2018): there was a lack of personal and professional experience of SM in general, compounded by a lack of institutional and professional guidelines. When guidelines were consulted they were often described as being ‘*purposively vague’*, providing little direction in terms of decision-making. This meant that many REC member interviewees felt they did not possess sufficient expertise to review and comment on SM research. This was in spite of some interviewees having consulted the literature or having taken some training to aid with their decision-making (Swift et al. 2018).

Without useful guidelines, ethical decision-making was performed on a case-by-case basis (‘*it’s very much on a case-by-case’* (interviewee 11)).

We are aware that this [their revised best practice guidance for research which uses social media data] was quite vague at the moment still. We tried to address the most significant issues that have come up, but because each case is different we can’t say “well, you have to do this every single time” because it might not be appropriate depending on the subject area or the methodology or what people want to do (interviewee 17).

Within this framework of ethical decision-making, focus was placed on researchers’ justifications of their research approach, including their rationale for chosen research questions, methodologies and publication practices:

I think it depends on the project…you can’t make blanket judgement and I think that’s why we look at applications in detail in each case. And sometimes make different decisions even for projects that look pretty similar. It’s how they build up their case doing that particular project (interviewee 15).

Without any accepted guidance, and with inconsistencies in REC interviewees’ approaches to ethical practice (Hibbin et al. 2018), researcher justifications of their research approach - including their rationale for chosen research questions, methodologies and publication practices - rather than standards, were often used to drive the process of ethical decision-making. This allowed for researchers’ ‘personal ethics’ approach to move through the review process to the point of publication.

### External Level Governance: Publishing Houses and Journals

Even though there are a number of SM research guidelines and initiatives currently available for researchers and affiliated institutions, regulatory mechanisms at the publishing houses and journals we analysed were still varied. None of the five main publishing houses had any specific policies on the use of SM data in research. Rather, all referred to their affiliations with the Committee of Publishing Ethics (COPE), which on investigation, also had no specific policies. Only two journals (n=2/11) reported any specific guidance on how to deal with research using SM data (further analysis of their respective websites showed that only one had unambiguous policies requiring all research to have ethical approval or an exemption; the other only specified this to be the case for ‘human participant’ research, which is itself an interpretive term in SM research). Practices were different for other journals, which often had less stringent requirements for ethical approval, and often considered ethical issues on a case-by-case basis. For example, for three Editors, ethics approval was not a necessity for publication, but rather, and especially when the research was not drawing on public data, researchers were required to justify their practices within ethics statements, considered on a case-by-case basis. Four journals were slightly more obligatory, stressing that ethical approval was not generally required for aggregate data studies, but was for studies containing any potentially identifying material, though again emphasis was placed on context and topic. One Editor noted that the onus for reviewing ethical concerns associated with any manuscript lay with the peer-reviewers, and was not something they as an Editor paid attention to. These findings lean towards the importance of the different levels of the Ethics Ecosystem - researchers, peer reviewers and Editors - as key judges of ethical practice. However, in the case of SM research, they suggest that inconsistencies or even questioning of ethical practice resulting from a ‘personal ethics’ approach may not always be picked up at the point of publication, especially given that peer reviewers may not be looking at ethical aspects of the research and Editors may rely solely on researchers’ justifications of the research. This could potentially result in discrepancies at the point of publication in terms of which studies had sought ethical approval, or at least had felt the need to ethically justify their approaches within their published methodologies.

### External Level Governance: Bibliometric Analysis

Our analysis of 324 UK peer-reviewed articles that drew on SM data (173 qualitative studies versus 151 quantitative studies) showed that 25 (8%) of SM studies had explicitly stated that they had sought ethics review (either approved or exempt) and 65 (20%) studies contained at least one justification for why ethics approval was not required within the context of the study, when ethics approval had not been sought. These justifications ranged from very brief statements about the use of publically available data within the context of the study, to much more explicit and sometimes lengthy discussions of the ethical implications of using SM data for studying social phenomena. These findings are similar to those reported by Taylor and Pagliari (2017). Ethics approval (or exemption) was almost only declared for studies that drew on data from online forums (e.g., Facebook, MySpace, LinkedIn, comments boards, gaming platforms etc; 88% of all studies declaring ethics approval or exemption). In total, 234 papers (72%) had no mention of ethics approval, no justifications for lack of approval, or discussion of the ethical implications of SM research within the methodology at all. While not all studies necessarily explicitly declare ethical clearance, the findings still suggest that the proportion of explicit ethical clearance declaration and/or consideration, is low.

For studies that did not declare ethical approval, but which explicitly ethically justified their use of SM data within their methodological approach, 24% of Twitter studies, 22% of online forum studies, 15% of blog studies, and 14% of YouTube/vlog studies included justifications to some degree. Whilst these numbers seem relatively similar, a closer analysis reveals areas of inconsistency. For example, a similar proportion of Twitter studies ethically justified their use of SM as a data source compared to blog/online forum studies, but the former group (all of the Twitter studies) comprised a higher proportion of quantitative big-data studies than the latter group (blog/online studies) (71% vs. 30%/37%), and therefore had different ethical risks attached to the research methodologies: aggregated, big-dataset analyses lower the risk of participant identification and qualitative non-aggregated data has a greater likelihood of participant identification due to the use of direct quotations. The reason for these inconsistencies is unknown.

These findings suggest that the ‘personal ethics’ culture could have prevailed through to the external level of the Ethics Ecosystem creating inconsistencies in ethical behaviour here, though further research would be required to confirm this. Moreover – and we return to this in the discussion - many researchers do not declare ethics approval or engage with ethics considerations (Hutton and Henderson 2015).

## Discussion

This paper has examined the promotion of ethical research behaviour on the network- rather than the individual actor-level for SM research via an analysis on the UK research Ethics Ecosystem. As Provan and Kenis (2007) state, ‘*networks…are more than the sum of the actors and their links and … deserve to be studied in their own right’* (Provan and Kenis 2007: 233). Ordinarily, the Ethics Ecosystem works because all members of this network participate equally and trust each other in the promotion, evaluation and enforcement of a shared understanding of ethically responsible research behaviour. We have shown some preliminary evidence that when a new research methodology such as health-related SM research enters the Ethics Ecosystem, and when the ethical issues related to said methodology are ambiguous, the network can potentially become imbalanced and inefficient. Specifically, we have shown some evidence that in UK SM research, where little shared overarching understanding of the ethics of this methodology has been developed, and guidelines are non-prescriptive, decisions about the ethical use of SM data - at least in the cases we have explored - is made by a reliance on individual researchers implementing a form of ‘personal ethics’. We recognise that further research would be required to confirm the prevalence of these findings.

The implementation of a ‘personal ethics approach’ is not necessarily problematic, since often it is researchers themselves who are at the forefront of new technological advancements and are best placed to consider and understand the ethical ramifications of their research. However, as we have shown in this paper, a ‘personal ethics approach’ can be equally problematic. First, it places the burden of ethical decision-making on researchers, who have to navigate the ethical terrain alone. This has left many researchers calling for more and appropriate guidelines, as we have seen is the case with SM research (Swift et al. 2018). Second, it leads to inconsistencies in terms of ethical decision-making and/or the need for ethical review (Hutton and Henderson 2015). Whilst, given the pluralistic nature of ethics, differences in ethical decision-making are not necessarily problematic, as research institutions it is important that we have a standard institutionalised approach to ethics, not least so that we can be seen to be both publically accountable and trustworthy. Third, a personal ethics approach which lacks shared understanding of ethical research within the Ethics Ecosystem may inadvertently allow some ethically problematic research to ‘fall between the cracks’ because the networked governance system is working inefficiently, as we saw in one example in the findings. And finally, a personal ethics approach means that much ethical decision-making is being led, and then re-enforced, by researchers themselves, who have a vested interest in conducting the research. Moreover, our preliminary findings suggest that with UK researchers by-passing ethical review, at least for the REC members we interviewed, they remain unaware of much of the research being conducted, remain inexperienced in the area, and fail to develop the tacit knowledge required to help them with their own ethical judgements about such research. If this situation is widespread, it potentially bypasses the REC as an important actor of the governance network and Ethics Ecosystem, and as a mediator of ethical research practice. Such a lack of REC member experience and expertise in negotiating ethical issues associated with new research methodologies has also been reported in other areas (Dove and Garattini 2017; Goodyear-Smith et al. 2015), and we have discussed this in more detail elsewhere (Swift et al. 2018).

Our pilot research is only hypothesis generating and cannot tell us how much we need to be concerned with the personal ethics approach identified in our findings, nor the prevalence of UK REC member inexperience – more research will need to be conducted in order to confirm this (for example, one UK study found little inexperience in SM research ethics review, though the findings were based on just one institution (Carter et al. 2016)). Having said this, our findings did show clear evidence of not only inconsistent practices in terms of SM research ethics governance, but also guidelines which were viewed by interviewees as vague and unhelpful. There was at least some evidence of a ‘personal ethics’ approach. To create a shared understanding of ethical behaviour by actors within the SM research Ethics Ecosystem so as to allow it to function correctly as a governance network, Provan and Kenis (2007) explain that when governance networks are in a state of flux they need to evolve and ‘*a specific choice must be made by network participants and managers to turn network governance over from one or more network participants to a third party organization’* (Provan and Kenis 2007: 247). In other words, when a governance network is no longer able to govern efficiently for whatever reason, the network must make a conscious decision to change the nature of its governance. This is most often and most efficiently achieved through the introduction of an external governance organisation. The UK SM research Ethics Ecosystem is an informal governance network based on trust, which means there is no immediate external organisation to take on this role. However, we argue that RECs could fulfill this role, as objective assessors of research integrity. This suggests that research using SM data could pass through an ethical review process – at least until there is a clearer shared understanding of the norms and standards of ethical practice for SM research.

Whilst this seems a feasible approach to address the preliminary findings of this paper, there are issues - the most prominent being that this would require *all* SM research to pass through ethics review, including those studies which only include a quantitative, big data, text mining-type approach. For these studies, which can be viewed as having little in the way of ethical connotations, ethics review adds an extra layer of bureaucracy. It can also be argued that compulsory ethics review such as this adds an additional ‘power’ element between researcher and REC/academic publisher, the latter whom would now require researchers’ to be more transparent about their methods if they wish to conduct their research, or have their research published (Foucault 2009).

The alternative to this would be discriminating between non-ethically problematic quantitative studies and other SM studies. However, this in itself raises issues related to what we are discriminating against (qualitative/quantitative; size of study; topic of study?) as well as questions about what we view as ethically problematic. Such decisions require further thought which, unfortunately, we do not have space for in this paper.

What we would like to note here is that the role of the REC in reviewing SM research should not take away the importance of the researcher. Researchers are important actors within the Ethics Ecosystem and should use the REC to support their decision-making - working together, both actors can discuss their knowledge and perspectives and learn from each other. RECs after all, are supposed to be a collaborative, supportive, bi-directional, longitudinal actor in the governance network, rather than a hierarchical gatekeeper.

Requiring (all) UK research to pass through REC review will ensure an institutional ‘ethical’ environment from which the generation of knowledge and learning, and eventual standards of practice can emerge. Whilst we note that RECs may deem much SM research exempt from review, by researchers asking the question it provides RECs with knowledge about the type of SM research being conducted in the institution, and what types of issues may arise from it. This is particularly important given the fast-changing nature of SM platforms, and in turn, associated research methods, which may raise new and different ethical issues over time [for example, the changing landscape of big data is making the distance between a person and their anonymised data closer together, raising additional questions about privacy (Hibbin et al. 2018)]. Having this broad overview then places RECs in a prime position to discuss and form consensus on the most appropriate ethical guidelines for SM research. Such consensus may then require certain SM research to be exempt from ethical review, but this will be a consistent approach to standardisation. Moreover, such consensus may form a standard for all SM research ethics, or may eventually cease viewing SM research as a field in its own right in need of a common set of standards and ethical obligations, but rather embrace the view that SM data is just another new methodology to be integrated within each discipline and/or analytical practice, and have discipline/analytical specific ethical guidelines. A discipline- or analysis-specific approach to SM research was emphasised in our interview data, and could be a better way to ensure that, rather than having all encompassing guidelines useful to no-one (and therefore providing a governance environment which permitting a ‘personal ethics’ approach), having more, but more specific guidelines useful to everyone (Cribb et al. 2008).

Whilst such a (temporary) change in governance may not solve all concerns related to how to conduct SM research ethically, we argue that, as a governance network based on shared understanding and trust of all actors, it is the most efficient approach for actors to learn, and to ensure a balanced UK Ethical Ecosystem of SM research.

## Footnotes

1 For example, see https://www.britsoc.co.uk/ethics.

2 http://journals.plos.org/plosone/article?id=10.1371/journal.pone.0127502.

3 Not all researchers’ beliefs about ethical review will affect their practices since some institutions demand ethical review for SM health research. However, as we show below, institutional practices are inconsistent and under-developed, making it easier for researchers’ personal views about whether to receive ethical approval manifest in research practice.

4 http://www.esrc.ac.uk/funding/guidance-for-applicants/research-ethics/our-expectations-of-ethics-review/.
